# Accuracy, Precision, and Trending Ability of Electrical Cardiometry Cardiac Index versus Continuous Pulmonary Artery Thermodilution Method: A Prospective, Observational Study

**DOI:** 10.1155/2017/2635151

**Published:** 2017-10-09

**Authors:** P. B. W. Cox, A. M. den Ouden, M. Theunissen, L. J. Montenij, A. G. H. Kessels, M. D. Lancé, W. F. F. A. Buhre, M. A. E. Marcus

**Affiliations:** ^1^Department of Anesthesiology and Pain Management, Maastricht University Medical Center+, Prof. Debyelaan 25, 6202 AZ Maastricht, Netherlands; ^2^Department of Anesthesiology and ICU, St. Antonius Ziekenhuis, Postbus 2500, 3430 EM Nieuwegein, Netherlands; ^3^Department of Anesthesiology, University Medical Centre Utrecht, Heidelberglaan 100, 3584 CX Utrecht, Netherlands; ^4^Department of Anesthesiology, ICU and Perioperative Medicine, HMC, P.O. Box 3050, Doha, Qatar

## Abstract

**Introduction:**

Evaluation of accuracy, precision, and trending ability of cardiac index (CI) measurements using the Aesculon™ bioimpedance electrical cardiometry (Aesc) compared to the continuous pulmonary artery thermodilution catheter (PAC) technique before, during, and after cardiac surgery.

**Methods:**

A prospective observational study with fifty patients with ASA 3-4. At six time points (T), measurements of CI simultaneously by continuous cardiac output pulmonary thermodilution and thoracic bioimpedance and standard hemodynamics were performed. Analysis was performed using Bland-Altman, four-quadrant plot, and polar plot methodology.

**Results:**

CI obtained with pulmonary artery thermodilution and thoracic bioimpedance ranged from 1.00 to 6.75 L min^−1^ and 0.93 to 7.25 L min^−1^, respectively. Bland-Altman analysis showed a bias between CI_BIO_ and CI_PAC_ of 0.52 liters min^−1^ m^−2^, with LOA of [−2.2; 1.1] liters min^−1^ m^−2^. Percentage error between the two techniques was above 30% at every time point. Polar plot methodology and 4-quadrant analysis showed poor trending ability. Skin incision had no effect on the results.

**Conclusion:**

CI obtained by continuous PAC and CI obtained by Aesculon bioimpedance are not interchangeable in cardiac surgical patients. No effects of skin incision were found. International clinical trial registration number is ISRCTN26732484.

## 1. Introduction

Measurement of cardiac output (CO) and cardiac index (CI) is commonly used in patients undergoing cardiac surgery. The continuous pulmonary artery thermodilution technique is well known as a method for advanced monitoring of cardiovascular function and is regularly used as a clinical reference technique in method comparison studies. However, a couple of studies during the past years showed that a true golden standard for measuring cardiac output does not exist.

Due to its invasive nature, the use of the PAC is associated with severe risk and there is still discussion on whether the positive effects of PAC outweigh the adverse effects [1–5].

Today, there are less invasive or even noninvasive monitoring devices available. The ideal technique should be reliable, noninvasive, continuous, cost-effective, and user independent and should have a fast response time enabling rapid detection of hemodynamic changes [6]. Considering the growing age of the surgical population with severe comorbidity, it is likely that monitoring of CO will be important, also in non-cardiac-surgery patients. Moreover, assessment of CO and stroke volume (SV) is a prerequisite to establish early goal-directed therapy during the perioperative period.

One of the most recent noninvasive techniques for assessment of CO is based on a modified thoracic bioimpedance algorithm. Briefly, thoracic bioimpedance is based on the theory that the thorax is a blood filled cylinder. According to Ohm's law (resistance = voltage/current) this model assumes that the impedance of thoracic tissue is parallel to that of blood. Blood related impedance changes repeat themselves with every heart beat and are linked to cardiac activity [7]. During systole, approximately 60 milliseconds after opening of the aortic valve, erythrocytes change their position from a random alignment to one parallel to the axial blood flow. This results in an increased conductivity allowing estimating the acceleration of flow through the aortic artery. However, early adoption of the technique showed diverging results with respect to precision and accuracy [8].

The basic equation was modified by Bernstein and Osypka, so that the maximum rate of change of impedance is related to the peak aortic blood acceleration [9]. The method used in the Aesculon was initially described as electrical cardiometry and it contributes the increase in conductivity to the orientation change of the red blood cells to determine the velocity of the blood flow, claiming to be a more accurate technique in a wide spectrum of patient conditions and patient populations including neonates and children [10, 11].

The bioimpedance method shows good results in clinical studies in young healthy volunteers. However, reliability in critically ill patients and in perioperative use is not proven and the available literature is inconclusive [12–15]. Moreover, until now it is unclear whether interruption of the skins integrity by a surgical incision could be a source of error in bioimpedance measurements. Recently Huang et al. found indications that skin incision can interfere with the bioimpedance technique [16].

Most available studies were performed in a neonatal and pediatric populations or during cardiac surgery, mainly in the postoperative period. Studies in adult patients undergoing high-risk cardiac surgery are lacking; therefore, we studied patient in the entire perioperative period [10, 11, 17].

The aim of the present study was to compare the accuracy, precision, and trending ability of a thoracic bioimpedance technique with continuous pulmonary artery thermodilution before, during, and after surgical intervention.

Secondary aim was to assess whether interruption of the skins integrity and opening of the thoracic cavity by a surgical incision could be a source of error in bioimpedance measurements.

## 2. Materials and Methods

### 2.1. Study Design

The study protocol was approved by the institutional review board of the Maastricht University Medical Center+ (MEC 08-4-075) and written informed consent was obtained from each patient. In this prospective observational study, 50 adult patients planned for elective cardiac surgery were included. Exclusion criteria were age < 18 years and a contraindication for placement of a Swan Ganz catheter. As placement of a Swan Ganz catheter was needed to retrieve CO measurements, we only included patients receiving the PAC in accordance with routine care.

### 2.2. Measurement Protocol

After arrival in the operating theatre, patients were connected to standard monitoring including heart rate, invasive blood pressure, and oxygen saturation. Anesthesia was induced according to local protocols. Thereafter, after rubbing and cleaning the skin with alcohol to achieve a skin-to-electrode impedance as low as possible, four standard electrocardiogram electrodes were placed according to the manual of the Aesculon (Osypka Medical, Berlin, Germany) on the left part of the neck and on the left part of the thorax at the level of the processus xiphoideus. Then the Aesculon monitor was connected to the electrodes for continuous display of bioimpedance cardiac index (CI_BIO_). A PAC (Edwards Life Sciences Corporation, Irvine, CA, USA, continuous cardiac output VIP catheter with SvO_2_, model 746F8) was inserted via the right or left internal jugular vein in order to continuously measure thermodilution cardiac index (CI_PAC_). To exclude the possibility of incorrect measurements during rapid fluid injections and hemodynamic instability we measured CI_BIO_ and CI_PAC_ at hemodynamic stability, in the absence of engraving events (e.g., profound bleeding, hypotension, and arrhythmias) and during normothermia.

We used the mean of two measurements of continuous CO. We used this as result per measurement and presented the results for each time point separately. There was no correction for repeated measurements applied because we analyzed the results per time point separately and assumed the measurements to be independent due to the extensive fluctuations.

Measurements were performed at 6 time points (*T*): after induction and prior to incision (*T*1), prior to cannulation of the aorta (*T*2), 10 minutes after protamine administration (*T*3), 30 minutes after arrival in the ICU (*T*4), 1 hour after extubation (*T*5), and 1 day postoperatively at 08.00 a.m. (*T*6). In addition to CI, heart rate, arterial blood pressure, and central and peripheral temperature were recorded at these time points.

### 2.3. Statistical Analysis

A sample size calculation was performed based on a previous study by Schmidt and coworkers [18]. Assuming a true difference between CI_BIO_ and CI_PAC_ of 0.5 liters/min/m^2^ and corresponding standard deviation (SD) of 1.0 liter/min/m^2^, a total number of 33 patients were needed to reject the null hypothesis that the difference is 0 with a power of 0.8 and type 1 error of 0.05. To correct for loss to follow-up an additional 17 patients were included, resulting in 50 patients in total.

Data were checked for normality using the Shapiro-Wilk test histograms (visually), including the difference between CI_BIO_ and CI_PAC_. Descriptive analysis was performed using number (%) or mean ± SD. Differences between the absolute CI measurements were assessed using the paired *t*-test. Accuracy and precision of CI_BIO_ against CI_PAC_ at the various time points were assessed using Bland-Altman analysis and plots showing the bias, limits of agreement (LOA), and percentage error (PE) [19, 20]. CI_BIO_ and CI_PAC_ were considered interchangeable if the PE was <30%.

According to the literature, BMI might influence reliability of the bioimpedance measurements [18, 21]. Therefore additional analysis was performed comparing 16 patients with BMI > 30 with the remaining patients (BMI ≤ 30).

In the formulas for the LOA and PE, a *t*-statistic of 2.02 was used at the various time points (*N* = 50) and 1.97 for pooled data (*N* = 300). To evaluate trending ability, four-quadrant plot and polar plot methodology was applied to the change in CI_BIO_ and CI_PAC_ between the time points [22, 23]. Concerning polar plot analysis central zone data (<10% change) were excluded because they introduce statistical noise. Angular bias is defined as the mean polar angle to the 0° line. The radial LOA refer to the radial sector that contains 95% of the data points. Polar concordance represents the percentage data points that lie within ±30°. In case of good trending ability, most of the data points lie within this 30° sector [23, 24]. Trending ability of CI_BIO_ was considered interchangeable with CI_PAC_ if angular bias was between −5° and +5°, with radial LOA between −30° and +30°.

A *P* value < 0.05 was considered statistically significant and Bonferroni correction for multiple testing of the absolute differences at the six time points was applied (*P* < 0.05/6 measurements = 0.008).

Statistical analysis was carried out using SPSS software (SPSS Inc., Chicago, IL, USA) and Excel (Microsoft Corporation).

## 3. Results

Fifty patients undergoing cardiac surgery were included. The baseline characteristics are presented in Table 1. The hemodynamic variables and temperatures are presented in Table 2. CI varied between 1.00 and 6.75 (CI_PAC_) and 0.93 and 7.25 L min^−1^ (CI_BIO_). CI_BIO_ and CI_PAC_ were significantly different at each point except *T*6 (24 hours after surgery).

Differences were present at open chest (*T*2, *T*3) and closed chest (*T*5, *T*6), respectively, indicating noninterchangeability between both techniques at both open and closed chest.

**Figure 1:**
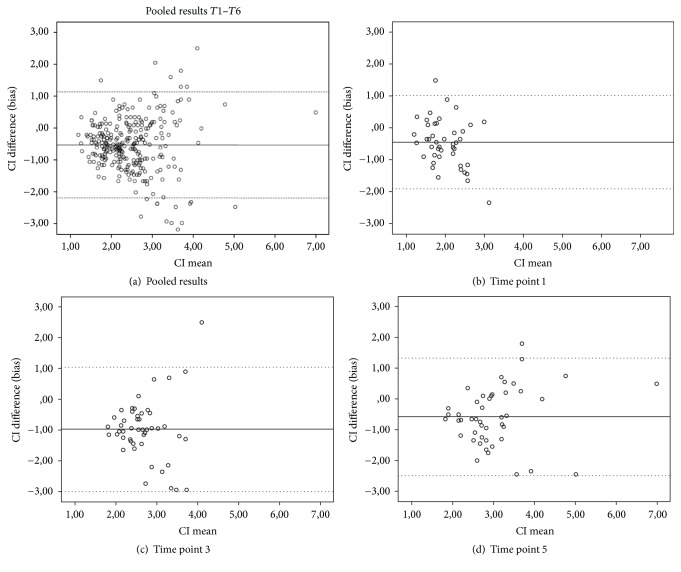
Bland and Altman plot. Fixed line indicates mean difference, and dotted lines indicate limits of agreement. (a) Pooled results, time point (*T*) 1 prior to surgery, after induction; *T*3  10 minutes after protamine administration; *T*5 one hour after extubation at the ICU ward.

Bias between CI_BIO_ and CI_PAC_ was 0.52 liters min^−1^ m^−2^, with LOA of [−2.2; 1.1] liters min^-1 ^m^−2^ (Table 3). Visual assessment of the Bland-Altman plots (Figure 1(a)) however shows that some agreement might be present at CI values between approximately 1.5 and 2.8 liters/min/m^2^. At higher CI values, the spread in the differences between CI_BIO_ and CI_PAC_ rapidly increases, especially at *T*1, *T*3, and *T*5 (Figures 1(a)–1(d)).

The percentage error between CI_BIO_ and CI_PAC_ was above the 30% agreement limit at every time point, including the lower limit of the 95% confidence intervals (Table 3).

The percentage error in patients with BMI > 30 ranged from 30% to 62% versus 52% to 79% in the patients with BMI ≤ 30.

**Figure 2:**
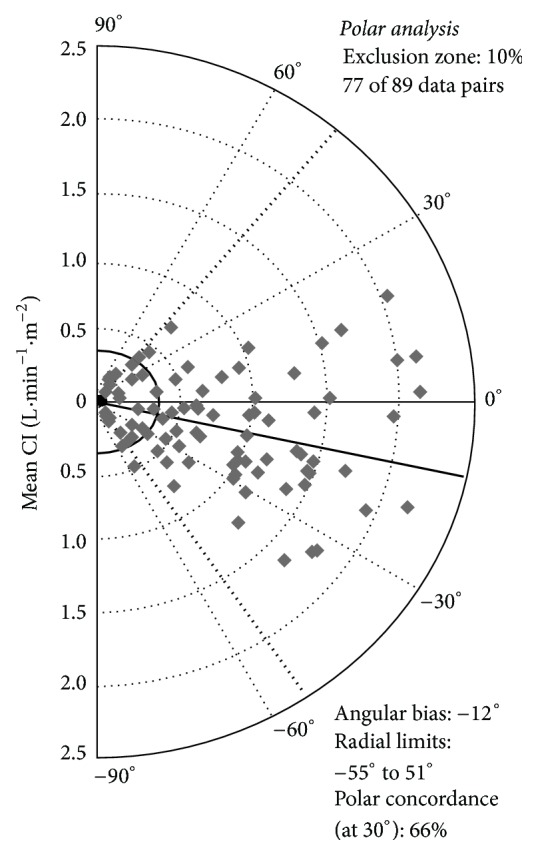
Polar plot analysis. In the polar plot, the distance from the centre represents the magnitude of the change in CI, whereas the angle with 0° line refers to its direction. Increases and decreases in CI are shown together in the so-called “half-moon” design. The mean change in CI (mean ΔCI) was used, with a 10% exclusion zone. Ideally, the mean angle of all data points is 0°, with at least 95% of all data points within the −30° to 30° sector (small dotted lines). Angular bias and radial LOA are depicted (solid line and thick dotted lines).

**Figure 3:**
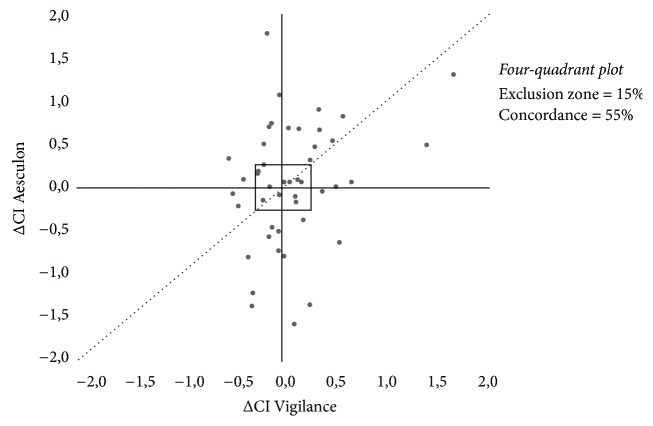
Four-quadrant plot analysis. Assessment of trending ability for pooled data. In the four-quadrant plot, the change in CI Vigilance™ (*x*-axis) is plotted against the change in CI Aesculon (*y*-axis). Ideally, all data points lie along the line of identity “*y*  1/4  *x*” (dotted line). The central square refers to the 15% exclusion zone. The zero axes “*y*  1/4  0” and “*x*  1/4  0” cross in the centre of the plot, creating four quadrants. In the right upper and left lower quadrants, CI Aesculon and CI Vigilance agree, which means that CI Aesculon and CI Vigilance change in the same direction. The concordance refers to the percentage of data points in these quadrants.

Trending ability was assessed in 89 pairs of changes in CI (Figure 2). Polar plot analysis in 77 data pairs outside the 10% exclusion zone showed an angular bias of −12°. The radial LOA were −55° to 51°. All values were outside the boundaries for acceptable trending ability. Polar concordance at 30° was 66%. These results were outside the boundaries for acceptable trending ability. Four-quadrant plot analysis also showed poor trending ability. Data pairs outside the 15% exclusion zone showed a concordance of only 55% (Figure 3).

## 4. Discussion

The present study investigated the accuracy, precision, and trending ability of a thoracic impedance CO monitor (Aesculon) versus pulmonary artery thermodilution in patients undergoing cardiac surgery. Our results do not support interchangeability of both devices in this patient group during surgery as well as during the early postoperative period, as both PE and trending ability exceeded the clinically acceptable, predefined limits.

Visual interpretation of the Bland-Altman plots revealed only moderate agreement at CI between 1.5 and 2.8 liters/min/m^2^. However, dispersion increased with higher CI. This so-called “heteroscedasticity” or proportional spread indicates that significant imprecision arises with increasing CO and that the LOA may even be underestimated in the high CO range. Several studies compared CO derived from bioimpedance with intermittent or continuous pulmonary artery thermodilution. The results of agreement and precision were inconclusive, demonstrating a need for additional studies in this field before drawing definitive conclusion on the validity and reliability of the Aesculon device technique [17, 25–27]. Patients undergoing cardiac surgery still represent a clinically challenging patient population, with relevant perioperative morbidity and mortality.

In comparable high-risk surgical patients, the use of invasive hemodynamic monitoring in combination with goal-directed therapy has been shown to improve postoperative outcome [28, 29]. In principle, thoracic bioimpedance represents a promising, noninvasive technique to be used in goal-directed strategies as the technique is noninvasive and independent from the observer and can be used also in patients at the ICU and medium care unit. Before implementation however, the reliability of the technology should be confirmed in the appropriate target patients. Apart from the clinical urgency, we decided to study patients undergoing cardiac surgery, because aortic cross clamping and clamp release induce profound changes in cardiac afterload followed by a profound ischemia-reperfusion injury. This implicates the necessity of profound observation and monitoring of these patients.

With the emergence of devices for continuous CO measurement, research increasingly focuses at trending ability rather than accuracy of individual measurements at a specific time point [30]. Especially during and immediately after cardiac surgery, the CO change in time may be more interesting than its absolute value, since the patients usually suffer from cardiovascular comorbidity and limited cardiac reserve [27, 28]. Therefore, 4-quadrant plot and polar plot methodology was used to objectify trending ability of the new technique. The results indicate poor trending ability, which was expected regarding the results from Bland and Altman analysis. Usually, high mean errors indicate that there is no fixed deviation between the experimental and reference technique, which impedes tracking of changes in CO in a reliable manner.

One of the strengths of the present study is the fact that most studies comparing the Aesculon with an established technique focus on neonatal, critically ill, and postoperative patient populations [10, 11, 15, 25–27].

Furthermore, there is lack of reliable published data on the trending abilities of the Aesculon [22]. In the present study, a relatively homogeneous population of cardiac surgical patients was studied during the intra- and postoperative period. As we were interested in the ability of the thoracic impedance under different conditions, we analyzed baseline values before surgery (*T*1) compared to open chest (*T*2, *T*3) and closed chest (*T*5, *T*6) conditions. However, the results demonstrated that the noninterchangeability between both techniques was present during both, open and closed thorax. In addition, we obtained CI measurements before and after weaning from mechanical ventilation and extubation up to 24 hours after surgery to simulate a medium care environment. We found the difference between both techniques independent from these conditions above the accepted range of 30%.

In our study population 16 patients had a BMI > 30. The percentage error was slightly lower in the BMI > 30 group but in both groups above the 30% limit. Clinical implication of these findings is questionable.

We included a substantial number of patients more than the calculated sample size to be sure we would have enough data. The concern was that we would have to deal with loss of data during observation at the ICU and possible failure of the Aesculon device. Accurate sampling at time points 5 and 6 also could be an expected problem.

Continuous pulmonary artery thermodilution was used as the reference technique. We used the mean of two measurements of continuous CO. Although thermodilution CO (TDCO) is considered as best acceptable clinical standard method for CO measurement, TDCO has an intrinsic variability, in particular during changing hemodynamic conditions and high CO [31]. Compared to the single bolus method, the continuous CO method has limited accuracy particularly during hemodynamic instability and hypothermia [31–33]. Being a combined measure of precision, the mean error may also be the result of variability of the reference technique [29, 34].

The pulmonary artery thermodilution catheter (PAC) has a precision of ±20% or less. The combination of two precisions of ±20% equates to a total error of ±28.3%, which is commonly rounded up to ±30% and is clinically acceptable [35, 36].

Moreover, the generally accepted range for bias (20%) and mean error (30%) are still a matter of discussion. The relatively large limit for bias was chosen to take possible hemodynamic changes into account. Depending on the clinical context, the limits can be defined more narrowly or even less narrowly. In general, it is advisable to use predefined criteria for acceptable bias and LOA in each method comparison study, since Bland and Altman analysis does not provide definitive answers. The same applies to the boundaries for trend parameters.

Our secondary aim was to assess whether the surgical incision, and therefore the interruption of the continuity of the skin of the thoracic cavity and opening of the cavity itself, could be an important factor in the reported discrepancy between the two instruments. Also, our study was not specifically designed to answer this question; we could not find any evidence for this hypothesis. The percentage error was always above 30%, being even above 70% in *T*1 and *T*3. Also the bias was very unpredictable, and the highest and the almost lowest value were measured during incision of the skin.

## 5. Conclusion

Analysis according to Bland-Altman shows that the Aesculon cannot be used interchangeably with the PAC in the operation theatre as a tool for beat to beat clinical decision making. We found no effect of skin interruption on accuracy, precision, and trending ability of the investigated technique.

## Figures and Tables

**Table 1:** Patient and surgery characteristics.

	Mean (sd)	Range
Age (years)	64.2 (10.6)	40 to 81
Height (m)	1.72 (0.1)	1.55 to 1.94
Weight (kg)	83.3 (17.5)	60 to 150
BMI (kg/m^2^)	28.2 (5.0)	19.3 to 46.3
		*n*
Sex m/f		35/15
CABG^*∗*^		31
AVR^†^		10
MVR^‡^		2
*CABG and AVR or MVR*		5
Other		2

**Table 2:** Haemodynamic data.

	*T*1	*T*2	*T*3	*T*4	*T*5	*T*6
MAP	71.7 (8.8)	70.2 (10.0)	70.9 (9.6)	75.8 (12.0)	74.7 (7.5)	79.6 (11.4)
HR	61 (11)	68 (17)	80 (11)	80 (12)	90 (14)	86 (12)
CVP	11 (4.6)	9 (4.6)	10 (4.0)	7 (3.3)	7 (4.6)	7 (4.7)
CI Aesculon	1.8 (0.5)	1.9 (0.7)	2.2 (0.8)	2.2 (0.7)	2.8 (1.1)	2.6 (0.7)
CI Vigilance	2.2 (0.7)	2.3 (0.7)	3.2 (0.7)	2.6 (0.5)	3.3 (0.9)	2.9 (0.7)
Temp central	36.1 (0.5)	35.9 (0.5)	36.3 (0.3)	35.9 (0.5)	37.7 (0.5)	37.4 (0.5)
Temp peripheral	30.9 (1.8)	32.1 (1.6)	33.3 (2.2)	32.9 (1.7)	34.7 (1.8)	34.3 (1.9)

Mean (sd). MAP: mean arterial pressure (mmHg), HR: heart rate (beats min^−1^), CVP: central venous pressure (mmHg), CI: cardiac index (liters min^−1^ m^−2^), and Temp: central and peripheral body temperature (°C). Time point 1 (*T*1) prior to surgery, after induction of anaesthesia; *T*2 prior to cannulation of the aorta; *T*3  10 minutes after protamine administration; *T*4  30 minutes after arrival at the ICU; *T*5 one hour after extubation at the ICU; *T*6 first postoperative day, 8:00 a.m. in the ICU ward.

**Table 3:** Agreement results.

	Pooled	*T*1	*T*2	*T*3	*T*4	*T*5	*T*6
*N*	284	47	50	48	46	46	47
Bias (L min^−1^)	−0.52	−0.44	−0.38	−0.98	−0.45	−0.57	−0.28
CI bias (L min^−1^)	−0.62 to −0.42	−0.66 to −0.23	−0.60 to −0.16	−1.27 to −0.69	−0.62 to −0.27	−0.86 to −0.29	−0.51 to −0.05
LOA (L min^−1^)	−2.2 to 1.1	−1.9 to 1.0	−2.0 to 1.2	−3.0 to 1.0	−1.6 to 0.7	−2.5 to 1.3	−1.9 to 1.3
CI lower LOA (L min^−1^)	−2.3 to −2.0	−2.3 to −1.5	−2.4 to −1.6	−3.5 to −2.5	−1.9 to −1.3	−3.0 to −2.0	−2.3 to −1.5
CI upper LOA (L min^−1^)	1.0 to 1.3	0.7 to 1.4	0.8 to 1.6	0.5 to 1.5	0.4 to 1.0	0.9 to 1.8	0.9 to 1.7
Percentage error (95% CI)	67 (60 to 73)	74 (56 to 92)	77 (58 to 96)	75 (56 to 93)	49 (37 to 62)	63 (47 to 79)	58 (43 to 72)

Bias: difference between CI Aesculon and CI Vigilance. CI: 95% confidence interval. LOA: limits of agreement. Time point (*T*) 1 prior to surgery, after induction; *T*2 prior to cannulation of the aorta; *T*3  10 minutes after protamine administration; *T*4 30 minutes after arrival at the ICVU ward; *T*5 one hour after extubation at the ICU ward; *T*6 on day one postoperatively at 8:00 a.m. in the ICU ward.

## Abbreviations

PAC: Pulmonary artery catheter
EC: Electrical cardiometry
CO: Cardiac output
CI: Cardiac index
ICU: Intensive care unit
*T*: Time point
SPSS: Statistical package for social science
SD: Standard deviation
B-A: Bland and Altman.

## Additional Points

*Key Messages.* The bioimpedance cannot be used interchangeably with the PAC during cardiac surgery.
